# Identification of *Mycobacterium tuberculosis* in Clinical Specimens of Patients Suspected of Having Extrapulmonary Tuberculosis by Application of Nested PCR on Five Different Genes

**DOI:** 10.3389/fcimb.2017.00003

**Published:** 2017-01-17

**Authors:** Azar D. Khosravi, Ameneh Alami, Hossein Meghdadi, Atta A. Hosseini

**Affiliations:** ^1^Department of Microbiology, School of Medicine, Ahvaz Jundishapur University of Medical SciencesAhvaz, Iran; ^2^Health Research Institute, Infectious and Tropical Diseases Research Center, Ahvaz Jundishapur University of Medical SciencesAhvaz, Iran; ^3^Aria HospitalAhvaz, Iran

**Keywords:** extrapulmonary tuberculosis, *Mycobacterium tuberculosis*, nested PCR, clinical specimens, gene

## Abstract

Definitive and rapid diagnosis of extrapulmonary tuberculosis (EPTB) is challenging since conventional techniques have limitations due to the paucibacillary nature of the disease. To increase the sensitivity of detection of *Mycobacterium tuberculosis* (MTB) in EPTB specimens, we performed a nested PCR assay targeting several genes of MTB on EPTB specimens. A total of 100 clinical specimens from suspected cases of EPTB were processed. Standard staining for acid fast bacilli (AFB) was performed as the preliminary screening test. Extracted DNAs from specimens were subjected to Nested PCR technique for the detection of five different MTB target genes of *IS6110, IS1081, hsp65kd, mbp64*, and *mtp40*. On performing AFB staining, only 13% of specimens were positive, of which ascites fluid (33.3%), followed by pleural effusion (30.8%) showed the greatest AFB positivity rate. We demonstrated slight improvement in yields in lymph node which comprised the majority of specimens in this study, by employing PCR targeted to *IS6110*- and hsp65-genes in comparison to AFB staining. However, the yields in ascites fluid and pleural effusion were not substantially improved by PCR, but those from bone and wound were, as in nested PCR employing either gene, the same positivity rate were obtained for ascites fluid (33.3%), while for pleural effusion specimens only *IS1081* based PCR showed identical positivity rate with AFB stain (30.8%). The results for bone and wound specimens, however, demonstrated an improved yield mainly by employing *IS1081* gene. Here, we report higher detection rate of EPTB in clinical specimens using five different targeted MTB genes. This nested PCR approach facilitates the comparison and the selection of the most frequently detected genes. Of course this study demonstrated the priority of *IS1081* followed by *mtp40* and *IS6110*, among the five tested genes and indicates the effectiveness of any of the three genes in the design of an efficient nested-PCR test that facilitates an early diagnosis of paucibacillary EPTB cases, which are difficult to diagnose with the available standard.

## Introduction

Tuberculosis (TB) is a serious disease which accounts a major global public health problem worldwide (Ates Guler et al., 2014). According to the (World Health Organization, 2014), *Mycobacterium tuberculosis* complex (MTBC) has infected nearly one-third of the world's population, and TB remains as one of the top ten leading cause of death in the world. Although the lung remains the common site of TB infection, there are increasing reports of extrapulmonary tuberculosis (EPTB) from all over the world, showing the potential spread capacity of *M. tuberculosis* (MTB) in many organs in the body (Hillemann et al., 2011; da Cruz Furini et al., 2013). EPTB comprises about 15–20% of TB cases and can comprise up to 50% of TB cases in human immunodeficiency virus (HIV)-infected individuals (Mehta et al., 2012). Therefore, early action to control the disease and intervention in the transmission chain, necessitates accurate diagnosis and appropriate treatment (Bollela et al., 1999; Lee, 2015).

Currently, nucleic acid-based technologies are used for the detection of MTB with very high sensitivity and specificity (Eisenach et al., 1990), and it can overcome some of the problems associated with the classical standard laboratory methods. Microscopic examination of acid-fast bacilli (AFB) although is rapid and cost-effective, have low sensitivity and specificity particularly in paucibacillary specimens, and culture, even though it is considered the gold standard due to its high sensitivity, requires several weeks to produce a result, and also comprises lower sensitivity for the detection of EPTB (Therese et al., 2005; da Cruz et al., 2011). Since there are many problems associated with performance of conventional diagnostic methods, various molecular biological tools like polymerase chain reaction (PCR)-based assays are developed for the reliable, early detection and speciation of mycobacteria in clinical specimens of EPTB. PCR is currently the preferred method for identification of EPTB, as this method is rapid and proved to be sensitive for the detection of bacteria in paucibacillary specimens (Chakravorty et al., 2005). The PCR technique, which can detect < 10 bacilli per milliliter of different biological specimens, is an useful tool for the diagnosis of MTB (da Cruz et al., 2011). However, a serious problem of detecting mycobacteria by PCR techniques, is the presence of PCR inhibitory substances which are found to be more associated with the EPTB in comparison to the pulmonary specimens. The presence of inhibitors is largely due to the carryover of substances in the sample preparation that interfere with the activity of the polymerase. Other investigators recommended the re-amplification after diluting out the inhibitors to improve the yield (Alli et al., 2011). Variation in sensitivity is one of the obstacles that prevent the full standardization of PCR technique in the laboratories of clinical analysis centers. To overcome this problem, PCR can be improved by a series of modifications to be able to detect the low number of MTB bacilli (Meghdadi et al., 2015). Alternatively, combination of two PCR techniques in the form of nested-PCR will increases its sensitivity and specificity in comparison to the conventional single PCR (da Cruz et al., 2011). The sensitivity of nested PCR compared to conventional single PCR, has already been well-documented in several recent and previous studies (Mishra et al., 2005; da Cruz et al., 2011). In the latter study, the sensitivity of nested PCR for the diagnosis of MTB, was increased in comparison to the conventional single PCR. In general, due to serious challenges associated with the diagnosis of EPTB, a careful selection of several target genes, is essential for increasing the chance of detection of MTBC in EPTB paucibacillary specimens (Nisha et al., 2012). Therefore, the present study was designed with the aim to detect MTBC in clinical specimens of patients suspected of having EPTB. For higher improvement of detection rate, nested PCR technique targeting five different genes of MTBC, was used to find out the frequency of EPTB in Ahvaz, Iran.

## Materials and methods

### Sampling and specimen processing

A total of 100 specimens were obtained from clinically suspected EPTB cases on the basis of imaging, clinical findings, histological and cytological observations, admitted to the university teaching hospitals, Ahvaz Jundishapur University of Medical Sciences, Iran, during 1 year period from April 2014 to April 2015. The initial proposal on the work was approved in the University high research and ethics combined committee and necessary permission was obtained for specimen collection (Ethics code.1615 dated 21th April 2014), and specimens were appropriately de-identified. The specimens type were: 26 lymph nodes, 19 bone biopsies, 19 wound specimens, 13 pleural effusions, seven urine specimens, four breast biopsies, three CSF, three synovial fluids, three ascites fluids, two colon biopsies, and one para thyroid biopsy. Standard staining for acid fast bacilli (AFB) was performed for the specimens as the preliminary screening test after the necessary specimens processing.

DNAs were extracted from the specimens by High pure PCR Template Preparation Kit (Roche-Germany). The extraction protocol for fluids and tissue specimens was the same except for performance of a pre-treatment step for tissues. In brief, 200 μl tissue lysis buffer and 40 μl proteinase K were added to completely squelched tissue section and incubated for 1 h at 55°C for fully digestion. The processed tissue specimens were then underwent DNA extraction according to kit provider instructions. The extracted DNA was stored at −20°C until PCR amplification.

### Nested polymerase chain reaction

Nested PCR targeting five different genes of *IS1081* (Taylor et al., 2007), *IS6110* (Marchetti et al., 1998), *Hsp65* (Telenti et al., 1993; Velayati et al., 2014), *Mtp40* (Marchetti et al., 1998), and *Mpb64* (Therese et al., 2005), of MTB was performed in two steps using the primers listed in Table 1. All primers were obtained from Bioneer Co., South Korea. In the first reaction a DNA fragment of desired base pair (bp) size for each gene was amplified using a pair of external primers; and in the second reaction, the first PCR amplicon served as template for the nested amplification of a fragment of certain bp within the initial sequence of the target gene using a pair of internal primers. The amplicon size of each gene in both reactions of nested PCR are listed in Table 1 adjacent to each target gene.

**Table 1:** **The primers used for five genes target of ***M. tuberculosis*** in nested PCR**.

**Gene**	**Primer sequence**		**Amplicon size (bp)**	**Annealing temperature**
*Is1081*	Outer primers:	TB-Q1: 5′CGAAGGAAATGACGCAATGA3′	476	47°C
		TB-Q2: 5′ CATCTCCGCAAAGCCTGGG 3′		
	Inner primers:	TB-B1: 5′ ACAGGCGAGCCCGGATCTGCTG 3′	248	58°C
		TB-B2: 5′ GTTCAGCTCGCTTGCGGCGCTG 3′		
*Is6110*	Outer primers:	K: 5′-ATCGTGGAAGCGACCCGCCAGCCCAGGAT-3′	220	63°C
		J: 5′-CGGGACCACCCGCGGCAAAGCCCGCAGGAC-3′		
	Inner primers:	MTB1: 5′ CCT GCG AGC GTA GGC GTC GG 3′	123	68°C
		MTB2: 5′ CTC GTC CAG CGC CGC TTC GG 3′		
*Hsp65*	Outer primers:	TB15: 5′-CGT AYG ACG AAG AGG CCC GT- 3′	470	53°C
		TB17: 5′-WAS GGR TCC TCS AGG ACS GC-3′		
	Inner primers:	TB11: 5′-ACC AAC GAT GGT GTG TCC AT-3′	439	47°C
		TB12: 5′-CTT GTC GAA CCG CAT ACC CT-3′		
*mtp40*	Outer primers:	PT1: 5′-CAACGCGCCGTCGGTGG-3′	396	54°C
		PT2: 5′-CCCCCCACGGCACCGC-3′		
	Inner primers:	PT3: 5′-CACCACGTTAGGGATGCACTGC-3′	223	53°C
		PT4: 5′-CTGATGGTCTCCGACACGTTCG-3′		
*Mpb64*	Outer primers:	Mpb1: 5′ TCCGCTGCCAGTCGTCTTCC 3′	240	54°C
		Mpb2: 5′GTCCTCGCGAGTCTAGGCCA 3′		
	Inner primers:	Mpb3: 5′ ATTGTGCAAGGTGAACTGAG 3′	200	58°C
		Mpb4: 5′AGCATCGAGTCGATCGCGGA 3′		

The final volume of amplification reaction for the first and second round of individual gene targets, was 25 μl and consisted of 0.2 mM each dNTP, 1.5 mM MgCl_2_, 0.5 μM of each primer, 1.5 U of Super Taq™ DNA polymerase (Roche, Germany), and 5 μl of template DNA. The PCR conditions were as: initial denaturation at 95°C for 5 min, followed by 30 cycles of denaturation at 95°C for 30 s, annealing of respective primers at an appropriate temperature (Table 1), for 30 s, extension at 72°C for 30 s, and final extension at 72°C for 4 min. In the second reaction (nested), the PCR product of the first round was diluted (100-fold for *IS6110, mpb64* genes and 200-fold for *IS1081, hsp65*, and *mtp40* genes) and used as a template in the second reaction (nested). The cycling program for nested reaction was the same as the first, except for the number of cycles which was increased to 35. The PCR products were analyzed by gel electrophoresis on 2% agarose in 1X TAE buffer containing 0.5 μg/ml ethidium bromide, and visualized by using the Gel Documentation System (Proteinsimp, USA).

SPSS software (SPSS Inc., no. 13) was used for data analysis. The generalized estimating equation (GEE) test was applied for investigating of correlation and statistical relationships between the applied methods.

## Results

In the present study, the analyzed clinical specimens were obtained from 46 male and 54 female patients, with the majority in the age group of 41–60 years. The lymph nodes were the most frequent specimens (26%), followed by bone biopsies and wound discharges, each 19%. Thirteen specimens (13%), were positive by AFB staining, while by application of nested PCR targeting 5 genes of MTB, 26 specimens (26%), showed positive results.

For setting up and optimizing the nested PCR, 10 specimens (pleural effusions, sputum, and CSF) from clinically TB cases confirmed by culture and biochemical tests and real time PCR, and 10 specimens from confirmed non-TB cases (colonies from non-mycobacterial bacteria including Pseudomonas, *E. coli*, and Staphylococci) were included in the study. These control specimens underwent the same processing steps as the tested specimens. All positive control samples showed positive results for all tested genes, while none of the negative controls showed any positive results in nested PCR with either gene. The overall positivity rate for tested genes were as: *IS6110* (22%), *IS1081* (24%), *hsp65* (21%), *mbp64* (12%), and *mtp40* (22%) (Table 2).

**Table 2:** **Comparison of nested PCR results among EPTB specimens with different gene targets**.

**No. of specimens**	**Nested PCR ***IS6110*** (123 bp)**	**Nested PCR ***Hsp65kd*** (439 bp)**	**Nested PCR ***IS1081*** (248 bp)**	**Nested PCR ***Mpb64*** (200 bp)**	**Nested PCR ***Mtp40*** (223 bp)**
11	+	+	+	+	+
4	+	+	+	−	+
2	+	+	+	−	−
1	+	−	+	+	−
2	+	−	+	−	+
1	+	+	−	−	+
2	−	+	+	−	+
2	−	−	+	−	+
1	+	+	−	−	−

**Figure 1:**
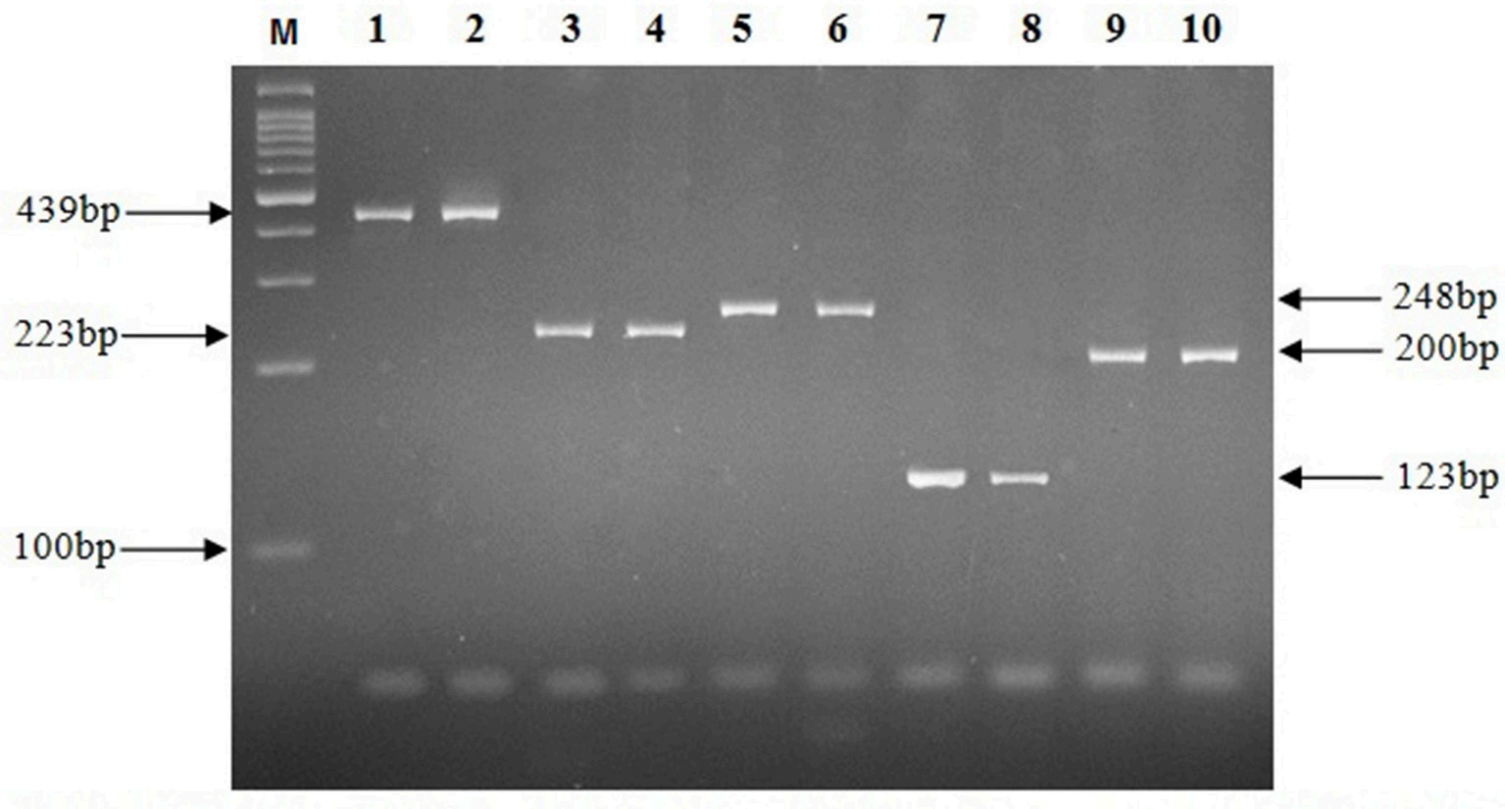
**Electrophoresis of PCR products on a 2% agarose gel**. M, Molecular size marker; Lanes 1, 2, 439 bp fragment of *hsp65* gene; Lanes 3, 4, 223 bp fragment of *mtp40* gene; Lanes 5, 6, 248 bp fragment of *IS1081* gene; Lanes 7, 8, 123 bp fragment of *IS6110* gene; Lanes 9, 10, 200 bp fragment of *mpb64* gene.

The results from AFB staining and nested PCR in relation to clinical specimens are presented in Table 3, and as it shows the highest positive rate for lymph node and CSF specimens, was obtained by *IS6110* (23.1%) and *hsp65* (33.3%) genes respectively. For bone (26.3%), and urine (14.3%) specimens, *IS1081* and *mtp40* genes showed more positive results, and in wound (36.8%), and plural effusion (30.8%) specimens, greatest positive rate was demonstrated with *IS1081* gene. Thus, the highest positive rate in nested PCR was achieved by *IS1081* gene (24%), while the least was reported for the *mpb64* gene (12%). For urine and CSF specimens, the positive results were achieved by only two out of five tested genes, and none of the breast, synovial fluid or colon specimens showed positive results by AFB staining or any of tested genes in nested PCR. Figure 1 represents the positive amplification results for target genes.

**Table 3:** **The overall positive rate of smear and nested PCR using five different gene targets of ***M. tuberculosis*** according to the type of extrapulmonary specimens**.

**Clinical sample**	**No. of samples**	**Positive smear (%)**	**Positive ***mtp40*** (%)**	**Positive ***mpb64*** (%)**	**Positive ***IS1081*** (%)**	**Positive ***hsp65kd*** (%)**	**Positive ***IS6110*** (%)**
Lymph node	26	4 (15.4)	5 (19.2)	4 (15.4)	5 (19.2)	6 (23.1)	6 (23.1)
Bone	19	0 (0)	5 (26.3)	2 (10.5)	5 (26.3)	3 (15.8)	4 (21.1)
Wound	19	3 (15.8)	6 (31.6)	3 (15.8)	7 (36.8)	6 (31.6)	6 (31.6)
Pleural effusion	13	4 (30.8)	3 (23.1)	2 (15.4)	4 (30.8)	3 (23.1)	3 (23.1)
Urine	7	1 (14.3)	1 (14.3)	–	1 (14.3)	–	–
Breast	4	–	–	–	–	–	–
Ascites fluid	3	1 (33.3)	1 (33.3)	1 (33.3)	1 (33.3)	1 (33.3)	1 (33.3)
CSF	3	-	-	-	-	1(33.3)	1(33.3)
Synovial fluid	3	–	–	–	–	–	–
Colon	2	–	–	–	–	–	–
Para thyroid	1	–	1 (100)	–	1 (100)	1 (100)	1 (100)
Total	100	13	22	12	24	21	22

In this study, a comparison made between the AFB staining and nested PCR results (Table 3). We demonstrated slight improvement in yields in lymph node, by employing PCR targeted *IS6110*- and hsp65 genes in comparison to AFB staining. However, the yields in ascites fluid and pleural effusion were not substantially improved by PCR, but those from bone and wound were, as in nested PCR employing either gene, the same positivity rate were obtained for ascites fluid (33.3%). While for pleural effusion specimens only *IS1081* -based PCR showed identical positivity rate with AFB staining (30.8%). The results for bone and wound specimens, however, demonstrated an improved yield mainly by employing *IS1081* gene.

Statistical analysis using GEE test on the results of nested PCR, revealed no significant differences in positivity rate for *IS6110, hsp65, IS1081*, and *mtp40* genes (*P* > 0.05), while significant differences were found between the positive rate achieved by *mbp64* gene in contrast to other tested genes (Table 4), and AFB staining results (Table 5).

**Table 4:** **Correlation significance between mbp64 gene with other target genes in nested PCR**.

**Variable**	**Beta**	**S.E**	**OR**	***P*****-value**
*IS6110*	0.727	0.229	2.07	0.001
*Hsp65*	0.668	0.241	1.95	0.006
*IS1081*	0.840	0.234	2.31	<0.001
*mtp40*	0.727	0.246	2.07	0.003

*SE, standard error; OR, odds ratio*.

**Table 5:** **Correlation significance between AFB smear results with results from nested PCR targeted to five different genes**.

**Variable**	**Beta**	**S.E**	**OR**	***P*****-value**
*IS6110*	0.635	0.229	1.89	0.006
*Hsp65*	0.576	0.198	1.78	0.004
*IS1081*	0.748	0.217	2.11	0.001
*mbp64*	0.091	0.241	0.91	0.705
*mtp40*	0.635	0.205	1.89	0.002

*SE, standard error; OR, odds ratio*.

## Discussion

The diagnosis of EPTB is always faced with challenges associated with sampling, as the collection of some of specimens such as plural effusion or bone biopsies requires invasive and laborious process. This usually leads to the lack of adequate specimen collection needed for processing. For this reason, a specimen should simultaneously undergoes various diagnostic tests such as histology, microbiology, biochemical analysis, and PCR. Sometimes, even in case of microbiologic analysis, the number of microorganisms in the specimen is inadequate or not distributed uniformly enough for robust detection. In the present work, for DNA extraction we used the high pure extraction and purification kit to minimize the inhibitors in EPTB. Moreover, a dilution of the product of the first reaction of PCR was used in nested PCR, which effectively reduced the concentration of the remaining inhibitory substances. However, since both false negative and positive results have also been reported for nested PCR (Sinha et al., 2016), employing several targets in PCR will improve the yields and eliminates the false positive or negative results which is more common in single target gene PCR performances. In the present study for elimination false results, we used the controls of confirmed TB and non-TB cases simultaneously. We obtained positive nested PCR results for all TB confirmed cases with all tested genes.

Several factors could influence the relative sensitivity of the target genes. The paucibacillary nature of EPTB specimens is a problem affecting the yield in PCR. The copy number of target genes in the genome of MTB is another matter. Although in molecular investigations of MTB, *IS6110*, and *IS1081* are commonly preferred target due to a higher copy number of these genes in bacterial genome, however in case of *IS6110*, there are reports of strains with low copy number (Sankar et al., 2011). On the other hand, in certain circumstances, a gene with under-limit copy number, could not be detected by nested PCR in spite of improvement of the technique.

Thus, in this study, by employing five various target genes of *IS6110, IS1081, hsp65, mbp64*, and *mtp40*, we tried to get more positive results and demonstrated 22, 24, 21, 12, and 22% positivity for EPTB specimens respectively. Nested PCR targeting *IS1081* gene, showed more sensitivity in comparison to other tested genes. Previously Fatolahzadeh et al. (2007), from Iran, used *IS1081*–PCR for the detection of pulmonary tuberculosis successfully and 78.2% of their isolates yielded positive results.

For CSF specimens, we obtained 33.3% positive rate by using either genes of *IS6110* or *hsp65*. Although the study of Sastry and Sandhya Bhat (2013) in India which was carried out on CSF specimens using *IS6110*-nested PCR, 40.3% positive rate was achieved which was higher than our findings, probably due to their larger sample size, as the number of CSF specimens in this study was too few to make any conclusion. Also Alfonso et al. (2002), in Colombia, reported a positivity rate of 74% by applying nested PCR targeting *mtp40* on clinical specimens (urine, sputum, CSF, etc.), while in present study 22% of the specimens were positive using *mtp40* genes. The reason may be due to inclusion of both pulmonary and extrapulmonary specimens in their work, rises the positive rate as work with pulmonary is much easier compared to extrapulmonary specimens.

Regarding *mpb64* gene as target in Nested PCR, we obtained 12% positivity rate. Therese et al. (2005), in India, reported 43.4% positive results using the same gene in nested PCR on various specimens suspected to EPTB, and they reported this gene as a sensitive target for the diagnosis of MTB in EPTB specimens. However, our findings, demonstrated the lowest positive rate by this gene representing lower sensitivity, compared to other target genes.

A comparison between the first and second reactions of nested PCR showed that results of the first reaction which is actually a representative of classic PCR, demonstrated lower positivity in comparison to second reaction for all tested genes, with the greatest positivity rate difference for *mtp40*, improving from 16 to 22%. Therefore, the results from this study demonstrated that nested PCR technique has higher sensitivity compared to traditional PCR method for the detection of MTB genome in specimens from EPTB. Our findings were concordant with other studies have emphasized on the superiority of nested PCR to conventional PCR in diagnosis of MTB (Marchetti et al., 1998; da Cruz et al., 2011). In this study, from total specimens of suspected EPTB, 26% of specimens were positive for at least one of the tested genes in comparison to confirmed TB and non-TB control specimens. Therefore, molecularly, the frequency of EPTB in our setting is determined as 26% in contract to lower frequency obtained by AFB staining (13%).

In conclusion, here we report higher detection rate of EPTB in clinical specimens using five different targeted MTB genes. This nested PCR approach facilitates the comparison and the selection of the most frequently detected genes. Of course this study demonstrated the priority of *IS1081* followed by *mtp40* and *IS6110*, among the five tested genes and indicates the effectiveness of any of the three genes in the design of an efficient nested-PCR test that facilitates an early diagnosis of paucibacillary EPTB cases, which are difficult to diagnose with the available standard methods. However, since for some specimen types such as CSF, ascites, or synovial fluids, the number of samples analyzed was low, more specimens in future studies will be needed to investigate the efficacy of target genes in the diagnosis. Moreover, a better comparison could be made with the results of phenotypic AFB staining.

## Author contributions

AK and AH: Substantial contributions to the conception, design of the work; Final approval of the version to be published; Agreement to be accountable for all aspects of the work in ensuring that questions related to the accuracy or integrity of any part of the work are appropriately investigated and resolved. AA: Acquisition, analysis, interpretation of data for the work; Agreement to be accountable for all aspects of the work in ensuring that questions related to the accuracy or integrity of any part of the work are appropriately investigated and resolved. HM: Acquisition, analysis, interpretation of data for the work; Final approval of the version to be published.

### Conflict of interest statement

The authors declare that the research was conducted in the absence of any commercial or financial relationships that could be construed as a potential conflict of interest.
